# Differences in Olivo-Cerebellar Circuit and Cerebellar Network Connectivity in Essential Tremor: a Resting State fMRI Study

**DOI:** 10.1007/s12311-022-01486-1

**Published:** 2022-10-10

**Authors:** Sarvi Sharifi, Arthur W. G. Buijink, Frauke Luft, Elliz P. Scheijbeler, Wouter V. Potters, Guido van Wingen, Tjitske Heida, Lo J. Bour, Anne-Fleur van Rootselaar

**Affiliations:** 1 Department of Neurology and Clinical Neurophysiology, Amsterdam UMC Location University of Amsterdam, Meibergdreef 9, D2-113, P.O. Box 22660, 1100 DD Amsterdam, The Netherlands; 2 Department of Biomedical Signals and Systems, University of Twente, TechMed Centre, Enschede, The Netherlands; 3 Department of Neurology and Clinical Neurophysiology, Amsterdam UMC Location Vrije Universiteit Amsterdam, Boelelaan 1117, Amsterdam, The Netherlands; 4 Department of Psychiatry, Amsterdam Neuroscience, Amsterdam UMC Location University of Amsterdam, Meibergdreef 9, Amsterdam, The Netherlands

**Keywords:** Cerebellum, Essential tremor, fMRI resting state, Connectivity, ICA

## Abstract

The olivo-cerebellar circuit is thought to play a crucial role in the pathophysiology of essential tremor (ET). Whether olivo-cerebellar circuit dysfunction is also present at rest, in the absence of clinical tremor and linked voluntary movement, remains unclear. Assessing this network in detail with fMRI is challenging, considering the brainstem is close to major arteries and pulsatile cerebrospinal fluid–filled spaces obscuring signals of interest. Here, we used methods tailored to the analysis of infratentorial structures. We hypothesize that the olivo-cerebellar circuit shows altered intra-network connectivity at rest and decreased functional coupling with other parts of the motor network in ET. In 17 ET patients and 19 healthy controls, we investigated using resting state fMRI intracerebellar functional and effective connectivity on a dedicated cerebellar atlas. With independent component analysis, we investigated data-driven cerebellar motor network activations during rest. Finally, whole-brain connectivity of cerebellar motor structures was investigated using identified components. In ET, olivo-cerebellar pathways show decreased functional connectivity compared with healthy controls. Effective connectivity analysis showed an increased inhibitory influence of the dentate nucleus towards the inferior olive. Cerebellar independent component analyses showed motor resting state networks are less strongly connected to the cerebral cortex compared to controls. Our results indicate the olivo-cerebellar circuit to be affected at rest. Also, the cerebellum is “disconnected” from the rest of the motor network. Aberrant activity, generated within the olivo-cerebellar circuit could, during action, spread towards other parts of the motor circuit and potentially underlie the characteristic tremor of this patient group.

**Supplementary Information:**

The online version contains supplementary material available at 10.1007/s12311-022-01486-1.

## Introduction

Essential tremor is a common movement disorder that characteristically reveals its tremor during action [1]. Tremor, which is mainly manifested in the upper limbs, is associated with aberrant oscillations within the motor system [2]. In the context of tremor, this network is also referred to as the tremor network [3]. In essential tremor, there is increasing evidence for abnormal functioning of one of the key entities of motor control, the cerebellum, that is hypothesized to be the origin of the aberrant oscillations within the tremor network [4–9].

The cerebellum has projections to the inferior olive, located in the brainstem, as well as the thalamus and motor cortices. The cerebellum, particularly its main deep cerebellar nucleus—the dentate nucleus (DN)—is known to play an important role in motor control [10, 11]. The DN mediates signals toward the contralateral midbrain, the ventral intermediate nucleus of the thalamus (VIM), and the sensorimotor cortex. Also, ipsilateral cerebellar cortices that are distributed according to a specific somatotopic organization are involved in sensorimotor integration, including lobule V of the anterior lobe and lobules VIIIA/B of the posterior lobe, whereas the remaining areas are thought to play an important role in non-motor functions [12, 13]. The inferior olive nucleus (ION) has also been mentioned as a key structure involved in the pathophysiology of tremor that is linked via the dentate-rubro-olivary circuit to the tremor network; however, evidence based on imaging studies is limited [14, 15].

Because manifestation of tremor in essential tremor is predominantly linked with voluntary movement, functional imaging faces a challenge of disentangling movement-related changes in brain activity from tremor-related changes in brain activity. Imaging studies often provoke tremor with a task-based protocol, thus activating areas involved in voluntary movement which are intertwined with the tremor network as well. Although the imbalances within the tremor network are only clinically visible during action, as action tremor, the underlying pathophysiological changes are presumably also present at rest. Also, during rest, brain activations will not be related to voluntary motor actions. For these reasons, resting state functional magnetic resonance imaging (fMRI) is of great value in the investigation of spontaneous (pathologic) neuronal activity in essential tremor.

Evidence from literature has already suggested resting state abnormalities in essential tremor. Earlier studies using positron emission tomography/single-photon emission computed tomography, while measuring regional cerebral blood flow (rCBF), demonstrated cerebellar hyperactivity at rest [16–19]. fMRI studies have furthermore indicated altered functional connectivity between the cerebellum and the rest of the tremor network and reduced effective connectivity within the cerebellum [20–28].

Previous studies mainly concentrated on regional homogeneity, and only a few made use of long-range connectivity within a priori–defined regions of interest (ROIs) [23, 29–31]. Also, studies mainly concentrated on functional connectivity implying correlations without investigating causal influences. Furthermore, pivotal areas (i.e., cerebellar structures and inferior olive) have not been investigated in detail. Investigation of these areas is challenging because of their small size, large inter-individual differences, and anatomical location close to major arteries and pulsatile cerebrospinal fluid–filled space [32]. Studies using tailored pre-processing methods investigating the olivo-cerebellar circuit in essential tremor are lacking.

The aim of the current study is to identify pathophysiological changes in essential tremor focusing on the olivo-cerebellar circuitry during rest compared to healthy controls. We will do this by (1) applying masked independent component analysis (mICA) enabling adequate data-driven analysis of the cerebellum/brainstem discarding notorious nuisance as much as possible [33], (2) evaluating functional and effective connectivity analyses within the cerebellum/brainstem based on identified components and cerebellar structures in literature that are known to contribute to motor control, (3) and finally, by investigating resting state whole-brain network connectivity within the tremor network with previously defined ROIs and whole-brain back projection of the cerebellar ICA components. We expect to find connectivity changes within the olivo-cerebellar circuit in ET compared to healthy controls compatible with an increased outflow of activity from the dentate nucleus and decreased connectivity between cerebellar and thalamo-cortical structures.

## Materials and Methods

### Participants

Patients with essential tremor diagnosed according to the criteria defined by the Tremor Investigation Group were recruited [1, 34]. Patients displaying bilateral postural arm tremor without other neurological disorders were included with an addition of a positive family history of tremor and a positive effect of propranolol on tremor. Seventeen essential tremor patients and 19 healthy controls without any known neurologic disorders were included. Patients were tapered off medication starting 3 days in advance to exclude confounding effects of medication. Tremor severity was assessed using the Tremor Research Group Essential Tremor Rating Scale (TETRAS) by an experienced movement disorder neurologist (JDS) [35]. All participants were right-handed according to the Edinburgh Handedness Inventory [36]. Exclusion criterion for both groups was cognitive dysfunction established with a Mini-Mental State Examination < 26. The study was conducted in accordance with the Declaration of Helsinki and was approved by the Academic Medical Centre Medical Ethical Committee. All participants gave written informed consent.

#### MRI Image Acquisition

The fMRI resting state protocol lasted 8 min. Participants were instructed to lie still with their eyes fixated on a cross, not to fall asleep, and not to think of anything in particular. Measurements were acquired with a 3.0 Tesla Philips MR scanner with a 16-channel head coil. Functional imaging parameters of the resting state scans were repetition time (TR), 2000 ms; echo time (TE), 30 ms; flip angle, 70°; voxel size, 3.5 × 3.5 × 3.5 mm; field of view (ap, fh, rl), 224 × 136.5 × 224 mm; and number of axial slices, 39. A structural T1 contrast MRI scan was obtained for registration and segmentation purposes with the following parameters: TR, 9 ms; TE, 3.59 ms; flip angle, 8°; slice thickness, 1 mm; voxel resolution, 1 mm × 1 mm × 1 mm; and number of axial slices, 170. Susceptibility weighted imaging (SWI) scans were also obtained for segmentation of the dentate nucleus (TR, 19.426 ms; TE, 25.48 ms; flip angle, 10; 1.2-mm slice thickness with 220 axial slices; and spatial resolution, 1.0 × 1.0 × 0.6 mm). The SWI sequence is a high-resolution 3-dimensional fully velocity–compensated gradient echo sequence. The iron content of the dentate nucleus is visible in the raw magnitude and phase images in individual subjects, improving localizing the dentate nucleus in individual subjects [37].

### Pre-processing of Resting State fMRI for Whole-Brain Analyses and Isolation of the Cerebellum

Data were pre-processed and analyzed using SPM12 (Wellcome Department of Cognitive Neurology, London, UK; https://www.fil.ion.ucl.ac.uk/spm) [38]. We applied a standard pre-statistics processing pipeline: realignment, slice-timing correction, co-registration with anatomical imaging, and spatial normalization to Montreal Neurological Institute (MNI) space. The spatially unbiased infratentorial template toolbox (SUIT, version 3.4) [39] was used for identifying activity maps of the cerebellum and brainstem. This high-resolution template allows exact subject-specific normalization of the cerebellum for accurate inter-subject alignment of cerebellar structures. The cerebellum for each participant was isolated from the anatomical whole-brain T1 scan, and a binary cerebellar mask was created and manually corrected if necessary (Supplementary Material Figure A). The cropped anatomical scans were normalized and resliced to the SUIT template with an isotropic voxel size of 1 mm. The full-width at half-maximum of the smoothing kernel for the whole-brain images was set at 8 × 8 × 8 mm^3^. For the cerebellar images, smoothing kernel was set at 4 × 4 × 4 mm^3^, considering the small size of cerebellar structures, to minimize spatial blurring. To achieve a steady-state signal, the first 5 frames of the functional images were discarded.

### Masked Independent Component Analysis

Functional network maps were derived using masked-ICA (mICA), making use of multi-session ICA with temporal concatenation as implemented in FSL Multivariate Exploratory Linear Optimized Decomposition into Independent Components (MELODIC) [33, 40–42]. With the help of SUIT masks, including the cerebellum and brainstem, 20 cerebellar resting state networks were identified using functional maps from both groups. The functionally relevant components were selected visually corresponding to the functional organization of the cerebellum and brainstem based on previous functional networks [13, 43–45]. To relate group averages back to individual participants and investigate whole-brain connectivity profiles of the mICA-derived local resting state networks of the cerebellum, a dual-regression technique was applied [46]. First, group mICA spatial maps were regressed onto the individual masked fMRI data sets, resulting in subject‐specific time courses for each IC. Second, these time courses were regressed onto the individual whole-brain functional data, so-called back projection. This produced subject-specific global connectivity maps of the five mICA components. This method was used previously in Moher et al. [40], where it proved to be a reliable method to assess brainstem and cerebellar resting state networks. Nonparametric permutation testing was performed to assess group differences (5000 permutations). The threshold for analyses was a *p* value of < 0.05 with family-wise error correction (FWE) using threshold free cluster enhancement (TFCE) (*p* value Bonferroni-corrected for amount of included *p* = 0.05/ICs) [47].

### Seed-Based Correlation Analysis: Functional Connectivity

**Fig. 1:**
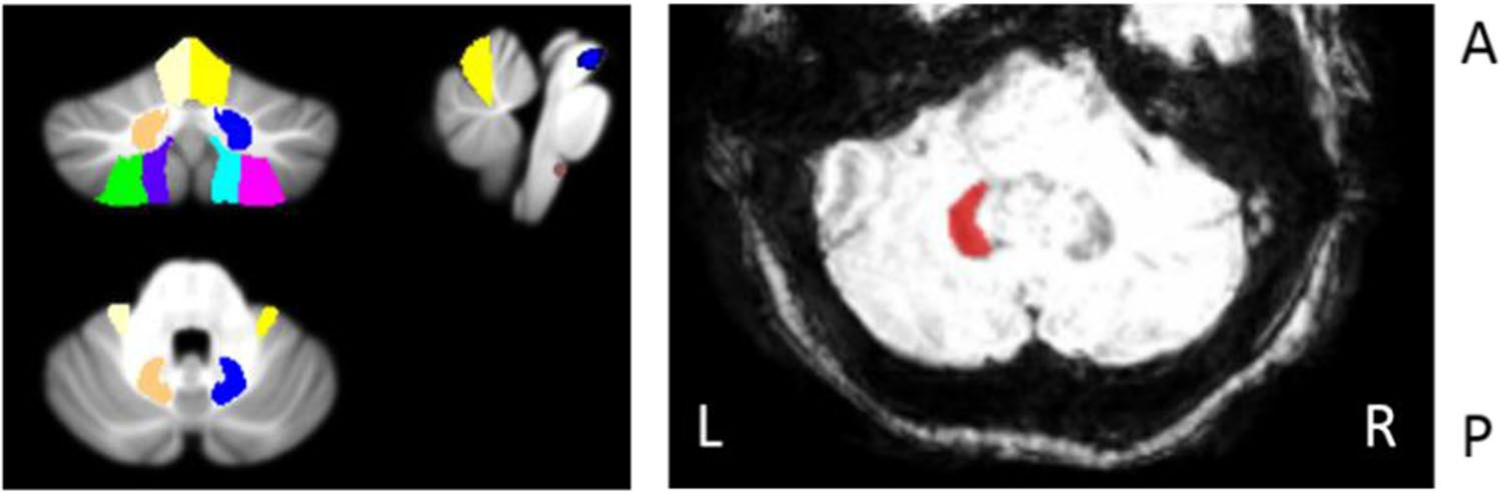
(Left) Regions of interest used for whole-brain (WB) analyses and intracerebellar analyses, including cerebellar lobule V (LV, yellow), cerebellar lobule VIIIa (LVIIIa, green), cerebellar lobule VIIIb (LVIIIb, purple), the dentate nucleus (DN, orange), red nucleus (RN, blue), and inferior olive of both hemispheres (IO, brown). All colors mentioned correspond to the left cerebellar hemisphere. (Right) An axial view of the SWI image of the middle area of the cerebellum. The SWI sequence is sensitive to high iron content, which can be used to identify the dentate nuclei as semicircular hypo-intensities, the opening facing the midline. In this figure, the left dentate nucleus is hand drawn onto the SWI scan of one participant

We used atlases in MNI space for determining seeds of interest. Cerebellar structures that are known to contribute to motor function were selected as seeds for further analyses [13]. For the functional connectivity analyses, seeds included the cerebellar lobule V (LV), cerebellar lobule VIIIa (LVIIIA), cerebellar lobule VIIIb (LVIIIB), and the dentate nucleus (DN) of both hemispheres. For the effective connectivity analyses, focusing on the olivo-cerebellar connectivity, we added the inferior olive (ION) and red nucleus (RN). The dentate-rubro-olivary circuit is linked to the cerebello-thalamo-cortical circuit via the cerebellum. Accurate anatomical determination of the dentate nucleus can be difficult because of its small anatomic size and varietal function. Therefore, personalized seeds of the dentate nuclei were derived from using SWI, where the deep cerebellar nuclei with high iron content are visualized as clear hypo-intensities [37, 48]. Time courses from the dentate nucleus were extracted before normalization, because ROIs were drawn onto the SWI scan of each participant in subject space. Mask of the inferior olive was based on the location reported previously [15, 49]; see Supplementary Figure B. Other seeds were extracted from functional images normalized to MNI space using the anatomy toolbox extension (AAL) included in SPM12 [50, 51]. The probabilistic atlases of the AAL and SUIT were used to identify anatomical locations of the activations [50, 52]. For the whole-brain analyses, images were spatially normalized to MNI space. For isolated brainstem and cerebellar analyses, images were spatially normalized to SUIT space, having more accurate inter-subject alignment of cerebellar structures. Figure 1 illustrates the regions of interest. Nuisance covariate regression was applied to reduce the impact of non-neural noise sources, including mean signal changes in the white matter, cerebrospinal fluid, and the six motion parameters [53]. Exclusion criteria for excessive motion were head motion with > 2.0-mm maximum displacement in any direction or 2.0° of any angular motion throughout the course of the scan.

We performed nonparametric permutation tests (Statistical Non-Parametric Mapping SnPM13.1.08, http://nisox.org/Software/SnPM13/, 5000 permutations). Main effects across groups are reported for voxels detected at *p* < 0.05 (if exceeding voxel count > 10), FWE-corrected for multiple comparisons within defined sensorimotor masks (sensorimotor cortex, basal ganglia, thalamus, cerebellum; Supplementary Material Figure C). Activity in regions showing significant functional connectivity differences was correlated with tremor severity (item A on the TRS) in the ET group.

### Effective Connectivity

**Fig. 2:**
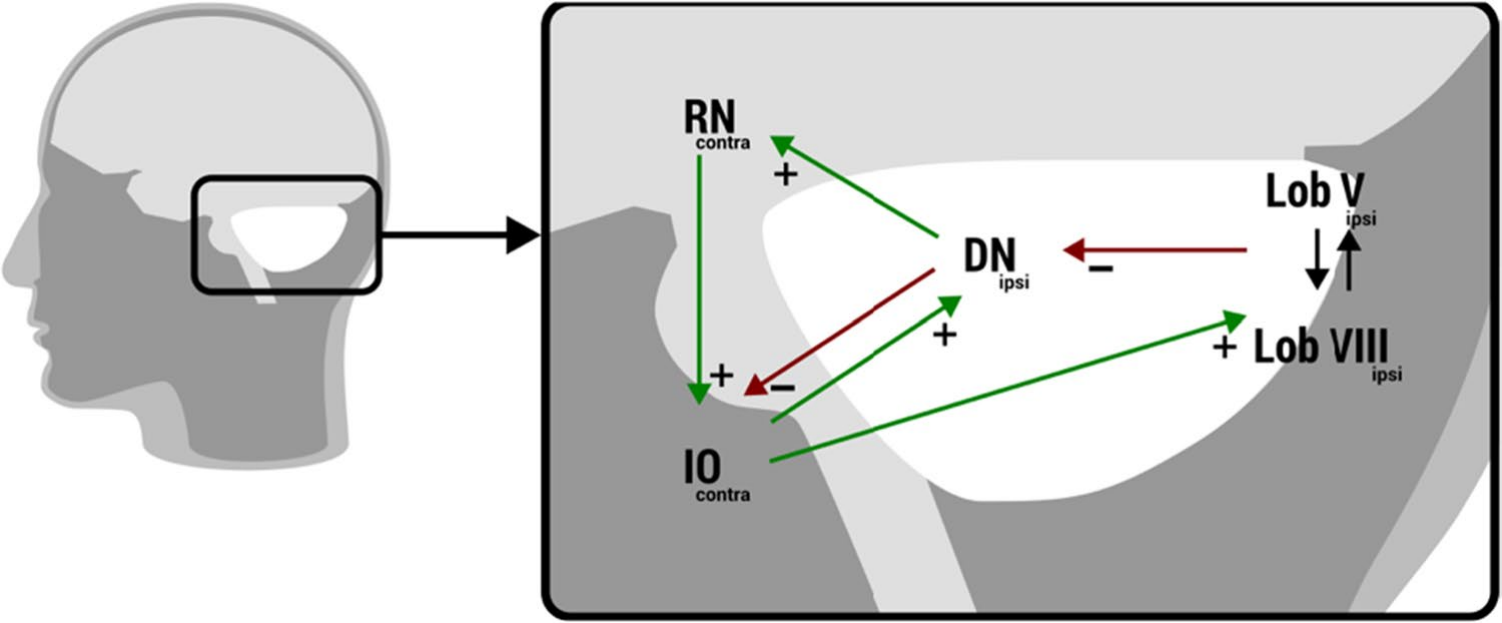
DCM modeled for the olivo-cerebellar network with ipsilateral cerebellar regions and contralateral red nucleus and inferior olive, depicted with its anatomical connections. For the inferior olive, a single mask, containing both the left and right nuclei, was used

Effective connectivity within the olivo-cerebellar network was investigated using spectral dynamic causal modeling (DCM; with the help of DCM12 in SPM12), an efficient method to estimate resting state directionality in the spectral domain [54]. DCM estimates the role of change in neural activity in one region due to dynamics in another region, resulting in two parameters: intrinsic within-region self-connection (A*I*) and extrinsic between-region connectivity (A*E*). Our proposed model was designed to investigate the olivo-cerebellar circuit and was based on anatomical connections. To investigate the specific connections between the inferior olive and cerebellum, we focused on the anatomical dentato-rubro-olivary circuit. This circuit is known to interact with the cerebello-thalamo-cortical circuit in the cerebellum. The dentato-rubro-olivary circuit includes connection with the ipsilateral dentate nucleus, contralateral red nucleus, and contralateral inferior olive. The proposed model is shown in Fig. 2, illustrated with its overall glutaminergic or GABAergic connections [55–58]. We analyzed the left and right circuits separately. In this study, the left circuit is defined as the left cerebellum and its connections with contralateral red nucleus and contralateral inferior olive nuclei. The right circuit is defined as the right cerebellum and its connection with the contralateral red nucleus and contralateral inferior olive nuclei. We used the same time series extracted for the functional connectivity and tested the left and right dentate-rubro-olivary circuits separately. The areas of interest are small, and capturing them using functional MRI techniques with limited spatial resolution is challenging, especially for the inferior olive nuclei in the medulla oblongata. For this reason, we used a single mask for the left and right inferior olive nuclei containing both nuclei.

Group-level differences of intrinsic and extrinsic connectivity parameters were assessed using a two-sample *t* test. Group-level analyses were only performed on remaining connections with estimated parameters that showed significant nonzero values in both groups determined with a one-sample two-tailed *t* test (*p* < 0.05).

## Results

All participants performed the resting state protocol without excessive movement as defined in the methods. All 17 essential tremor patients (4 women), with a mean ± SD age of 59.8 ± 17.4 years, were included. Also included were 19 healthy controls (7 women), aged 54 ± 12.3 years, without any known neurologic disorders. An overview of the participant characteristics is given in Table 1 (in detail Table I Supplementary Material).

**Table 1:** Participant characteristics; ET, essential tremor patients; HC, healthy control subjects; TETRAS, The Essential Tremor Rating Assessment Scale

Group		Gender #	Age (years)	Age at disease onset (years)	TETRAS
ET	Median (range)	M: 13	63.0 (22.5–84.5)	30 (< 18–60)	16.5 (11–36)
		F: 4			
HC	Median (range)	M: 12	59.40 (24.4–78.1)		
		F: 7			

First, we present the functionally relevant cerebellar resting state networks determined with mICA and their whole-brain back projections. Next, results of the functional and effective connectivity within the olivo-cerebellar areas are presented which are determined from the infratentorial toolbox (SUIT). At last, we present the whole-brain connectivity from the cerebellum to the other parts of the motor circuit (MNI atlas).

### Cerebellar Resting State Networks (mICAs) and Whole-Brain Back Projection

**Fig. 3:**
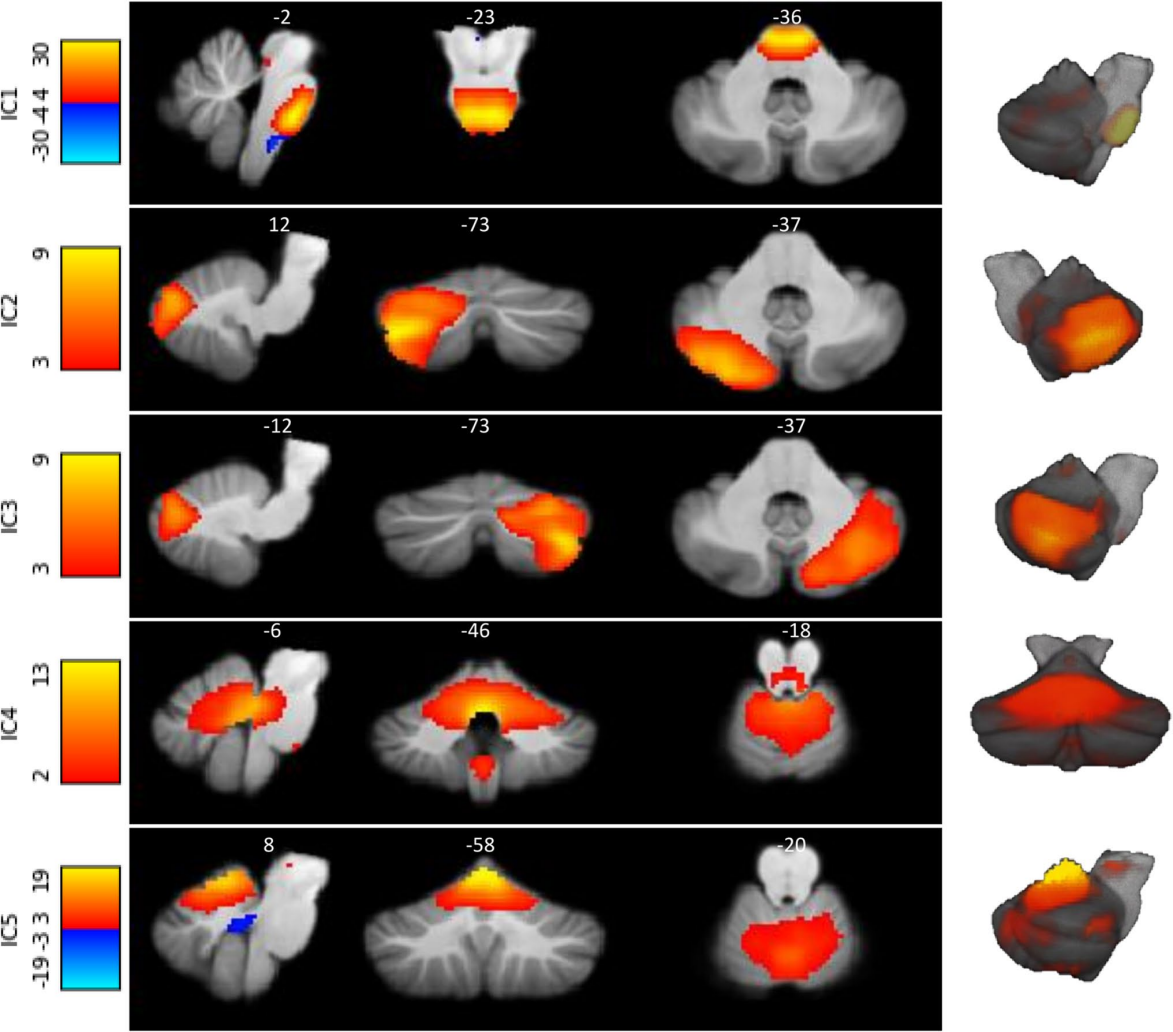
The five functionally relevant independent components based on both groups in resting state with cerebellar mask. Images are displayed in radiological orientation

The selected ICs representing local resting state networks in the cerebellum are shown in Fig. 3 and Table 2. We selected five functionally relevant components and labelled remaining 15 components as noise based on the fact that these were located at the borders between the cerebellum/brainstem and cerebrospinal fluid or did not correspond with known functionally relevant cerebellar clusters [43, 44].

**Table 2:** Results of the independent component analyses

IC	Cerebellar areas	Cerebellar resting state networks [43, 44]	Function [13]	Whole-brain back projection TFCE, FWEcor *p* < 0.05
1	Posterior lobe/Brainstem	Pons LIX L, LIX R, LVIIIb L, LVIIIb R, L I–IV R, L I–IV L	Connection	
2	Posterior cerebellum Right hemisphere	Crus I R, Crus II RLVI R, LVIIb R, LVIIIa R	Cognition	
3	Posterior cerebellum Left hemisphere	Crus I L, Crus II LLVI L, LVIIb L, LVIIIa R	Cognition	
4	Anterior/posterior lobe	Vermis VIIIa, Vermis VIIIa, LVI L, LVI R, LV L, LV R, Crus II L, Crus II R, DN LR	Cognition	
5	Anterior lobe	LV R, LV L, L I–IV L, L I–IV R Vermis VI, LVI R, LVI L,	Sensorimotor	Postcentral LR, Superior parietal lobule LR, Supramarginal LR

IC 1 comprised areas mainly in the brainstem (pons) and its connections with the cerebellum (lobules IX left and right, lobules VIIIB left and right, and lobules I–IV left and right). ICs 2 and 3 had a symmetric lateralized appearance, whereas all other components were more symmetric. ICs 2 and 3 encompassed, respectively, the right and left cerebellar hemispheres, mainly showing networks containing Crus I and II, which is associated with non-motor functions. IC 4 encompassed ventral structures related to the body of the vermis, including left and right lobules VI, V, Crus II, dentate nuclei, and the vermis VIIa, associated with non-motor functions. Finally, IC 5 comprised the left and right anterior and medial lobules, including cerebellar lobules I–VI, lobules V–VI, and the vermis of lobule VI, associated with sensorimotor functions.

**Fig. 4:**
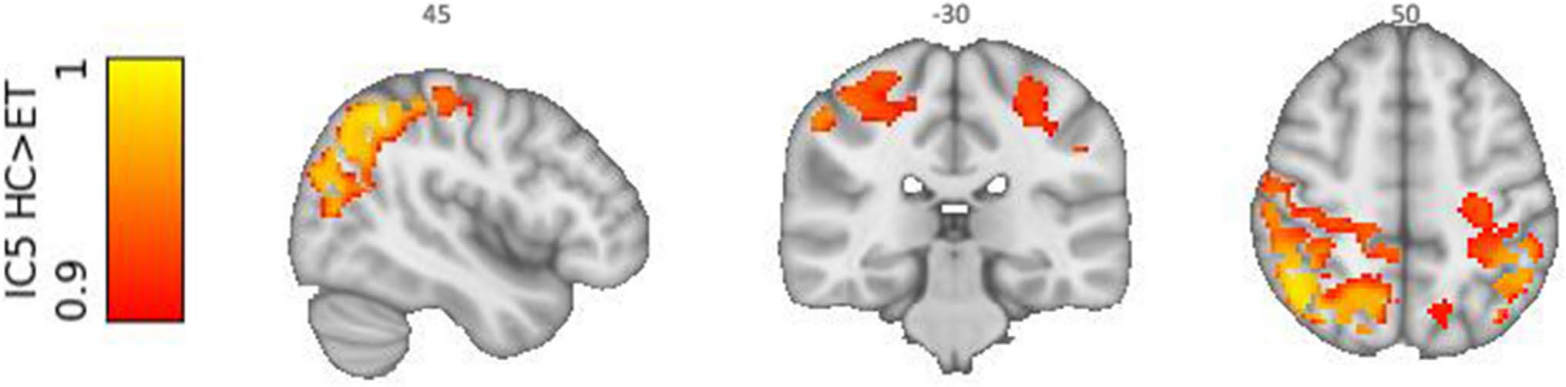
Group difference ICA after whole-brain back projection. Essential tremor (ET) patients compared to healthy controls (HC) show decreased connectivity with cerebellar IC5 (associated with sensorimotor network) and sensory cortices, including the primary sensory cortex and secondary cortical areas involved with somatosensory processing (*p* < 0.01 FWE-corrected). Depicted for visualization purposes FWE-corrected *p* < 0.1. Images are displayed in radiological orientation

The whole-brain connectivity of these components was evaluated on the group level. One whole-brain connectivity profile, based on the sensorimotor cerebellar resting state network (IC 5), showed significant differences between patients with essential tremor and healthy controls (whole-brain FWE < 0.01; Fig. 4).

Whole-brain back projection of IC5 showed in essential tremor patients decreased connectivity in mainly sensory cortices, including the primary sensory cortex and secondary cortical areas involved with somatosensory processing. The areas are listed in Table 2.

### Olivo-Cerebellar Functional Connectivity

**Fig. 5:**
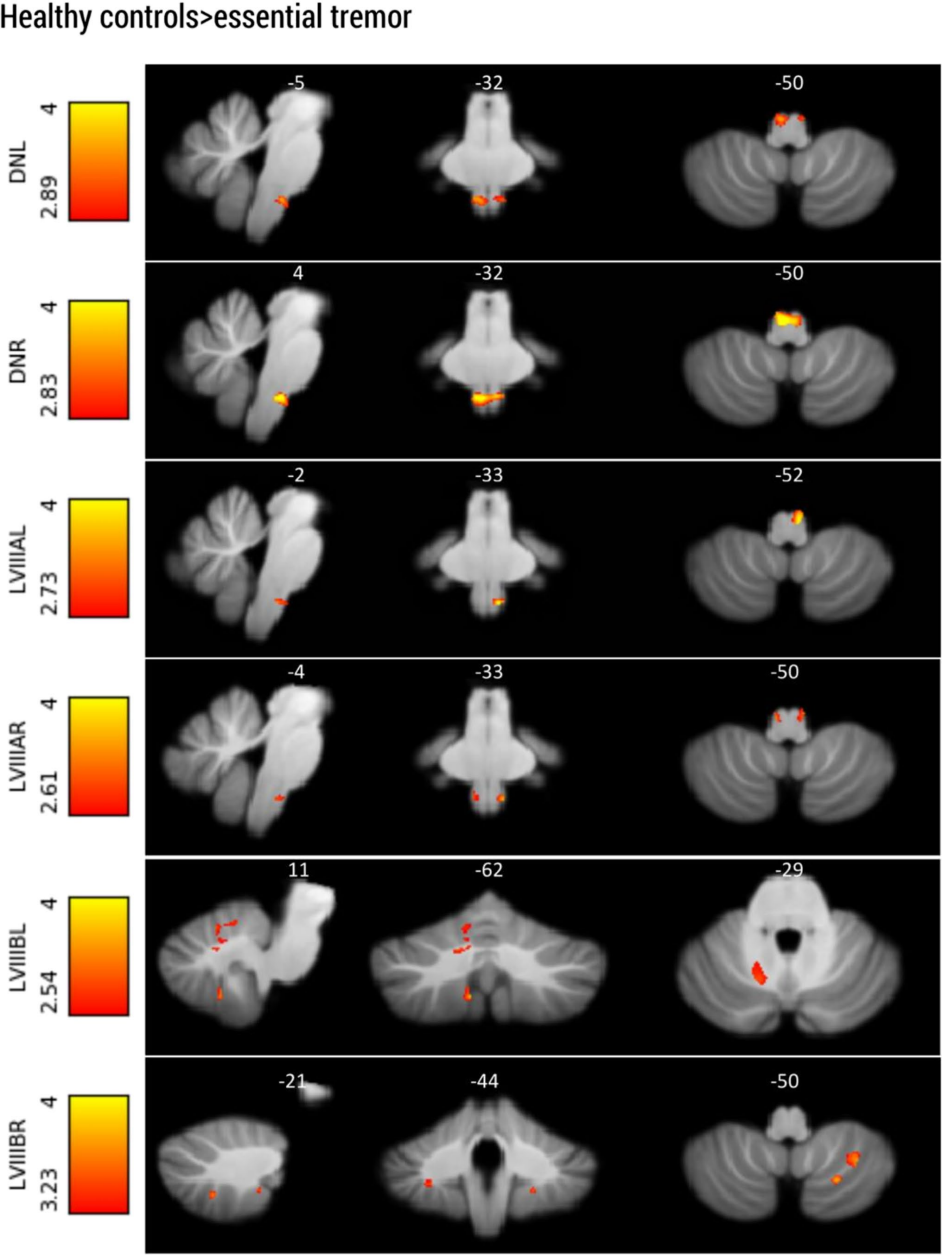
Significant functional connectivity differences between healthy controls and essential tremor patients in the olivo-cerebellar circuit, as detected by the SUIT analyses (*p* < 0.05, FWE-corrected). For the left and right dentate nuclei as well as the left and right LVIIIA, functional connectivity with the inferior olive nuclei was decreased. Furthermore, left and right lobules VIIIB show a decreased global connectivity with the cerebellum. Images are displayed in radiological orientation

A widespread decrease in connectivity was found between key cerebellar motor regions and the cerebellum/brainstem. As seen in Table 3 and Fig. 5, the essential tremor patients exhibited decreased functional connectivity between cerebellar ROIs and other areas of the cerebellum (i.e., the bilateral dentate nucleus, lobules V, lobules VIII). Essential tremor patients exhibited decreased connectivity between bilateral dentate nuclei and the inferior olive. The connection between the right dentate nucleus and the inferior olive correlated negatively with the tremor severity (TRS), FWE *p* < 0.03 T = 3.77. Cerebellar lobule V seeds did not show any significant clusters.

**Table 3:** Local maxima of group differences between essential tremor (ET) patients and healthy control (HC) subjects; VIM, ventral intermediate nucleus; LV, lobule V; LVIIIA, lobule VIIIA; LVIIIB, lobule VIIIB; DN, dentate nucleus; L, left; R, right

		MNI coordinates			Statistical tests		
Seed, contrast		*X*	*Y*	*Z*	*T*	*p*	Cluster size
DN left, HC > ET							
WB	ION	4	− 34	− 48	3.33	0.001	22
SUIT	ION	5	− 33	− 49	3.65	< 0.01	100
DN right, HC > ET							
WB	ION	− 2	− 34	− 48	4.82	< 0.003	48
SUIT	ION	5	− 34	− 49	4.62	< 0.001	288
LVIIIA left, HC > ET							
WB	Thalamus L	− 12	− 30	12	4.57	< 0.001	112
	Thalamus R	14	− 20	12	3.74	< 0.001	
SUIT	ION	− 5	− 32	− 53	4.17	< 0.001	66
LVIIIA right, HC > ET							
WB	Thalamus L	− 12	− 30	10	4.28	0.004	31
		2	− 18	− 10	4.31	< 0.004	44
	Thalamus R	18	− 30	10	3.89	< 0.02	58
SUIT	ION	− 6	− 34	− 51	3.56	< 0.004	45
		7	− 34	− 50	3.22	< 0.01	26
LVIIIB left, HC > ET							
WB	–						
SUIT	DNR	14	− 63	− 28	3.06	< 0.02	122
	LVIIIB R	9	− 62	− 50	3.86	< 0.02	34
	LV R	10	− 64	− 18	3.48	< 0.03	43
		18	− 64	− 18	3.42	< 0.03	181
		13	− 58	− 25	3.25	< 0.04	38
LVIIIB right, HC > ET							
WB	–						
SUIT	LVIIIAL	− 23	− 64	− 51	3.78	< 0.02	57
		− 32	− 57	− 51	3.76	< 0.02	78
	LVIIIAR	29	− 46	− 46	4.00	< 0.006	45
		13	− 65	− 45	3.71	< 0.002	84
	LVIIIBL	− 20	− 43	− 48	3.76	< 0.02	21
LVL/LVR							
WB/SUIT –

### Olivo-Cerebellar Effective Connectivity

**Fig. 6:**
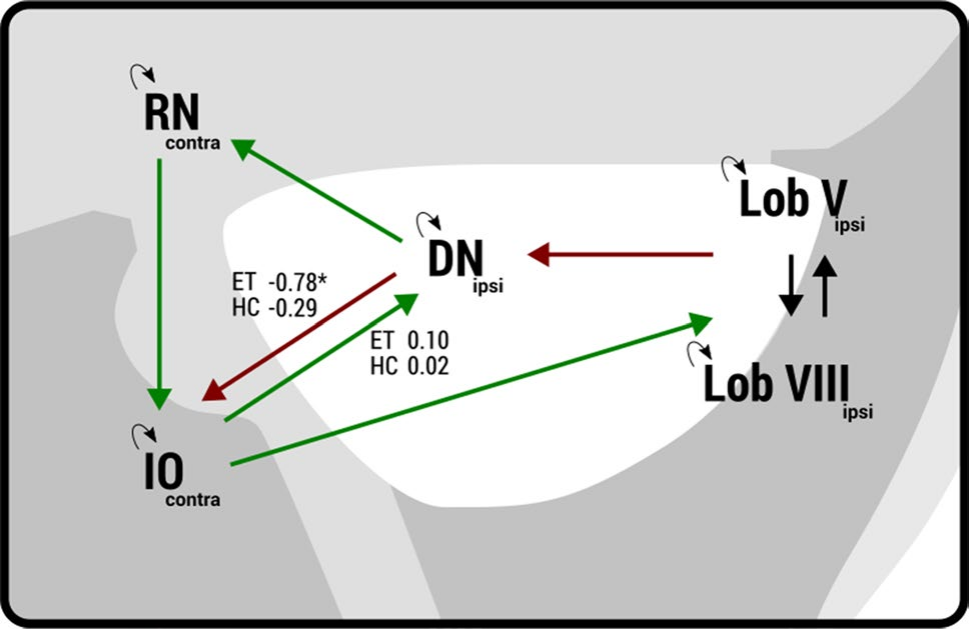
Parameters that show differences in effective connectivity between the dentate nucleus and inferior olive. Neural activity in the dentate nucleus has a more inhibitory effect on the inferior olive in essential tremor compared with healthy control group

Effective connectivity values were evaluated for the left and right cerebellar circuits (connected with contralateral red nucleus and inferior olive) separately. Extrinsic connections (between regions) of the left circuit showed a significant connection (significant deviation from 0) in the reciprocal connections between the dentate nucleus and inferior olive nucleus. In the left cerebellar circuit, the extrinsic connection from the dentate nucleus to inferior olive was significantly different (Fig. 6). As demonstrated in Fig. 6, the dentate nucleus modulates the circuit by having an increased inhibitory influence on the inferior olive in the essential tremor group compared with healthy controls (ET − 0.78 ± 0.53, healthy controls − 0.29 ± 0.76). The right cerebellar circuit showed the same trend but did not reach statistical significance (Supplementary Material Table II and III). There were no significant differences in the intrinsic connectivity parameters between the groups.

### Whole-Brain ROI Analyses in MNI Space

**Fig. 7:**
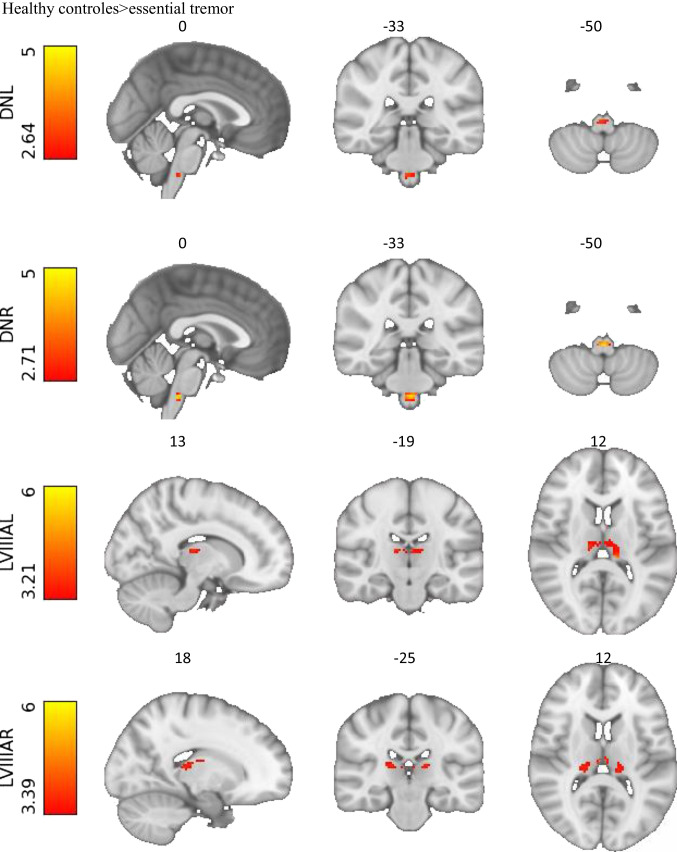
Significant functional connectivity differences between healthy controls and essential tremor patients in the cerebello-thalamo-cortical circuit, as detected by the whole-brain analyses (*p* < 0.05, FWE-corrected). Connectivity of the right and left dentate nuclei with the inferior olive is decreased. Furthermore, right and left lobules VIIIA show a decreased connectivity with the thalami. Images are displayed in radiological orientation

Essential tremor patients compared with healthy controls demonstrated decreased functional connectivity between both cerebellar lobules VIIIA and bilateral thalami. Bilateral dentate nuclei demonstrated decreased connectivity with the inferior olive in the medulla oblongata. The connection between the right dentate nucleus and the inferior olive correlated negatively with the tremor severity (TRS), FWE *p* < 0.03 T = 3.30. Connectivity between the dentate nucleus and the rest of the network did not differ between groups. Finally, no significant differences were found between the functional connectivity profiles of lobules V and VIIIB. Results are listed in Table 3 and displayed in Fig. 7.

## Discussion

The cerebellum is a pivotal structure in the pathophysiology of essential tremor [4, 29, 59–63]. Here, we show that previously defined key structures in the cerebellum, related to motor functions, demonstrate decreased connectivity with cerebral areas and the thalamus. Also, there is widespread decreased connectivity within the cerebellum and related brainstem structures, such as between dentate nuclei and inferior olive nuclei. Results of our data-driven approach using ICA and hypothesis-driven ROI analyses both lead to similar results. With ICA, we identified a local resting state network in the cerebellum and brainstem that is associated with motor functions. This cerebellar motor network shows, in contrast to resting state networks associated with non-motor functions, decreased connectivity with cerebral cortical areas. Our results support the hypothesis that tremor in essential tremor is the result of a pathologic disorganization within the olivo-cerebellar system.

### Disturbed Dentato-Olivary Connectivity: Disorganized Cerebellar Oscillations

In the intracerebellar SUIT analyses, and supported by the whole-brain analyses, bilateral dentate nuclei show decreased connectivity with the inferior olive. Other functional resting state studies have suggested functional alterations as well, including altered cerebello-thalamo-cortical connectivity [29–31]. Also, perfusion studies (PET/SPECT) have shown a hyperactive cerebellum at rest [16–19]. In addition, our results are the first to show altered dentato-olivary connectivity. The inferior olive has glutamatergic connections towards the cerebellum and receives inhibitory input from the cerebellum via γ-aminobutyric acid (GABA) receptors [55]. In rest, our functional connectivity results suggest decreased dentato-olivary connectivity in essential tremor. The effective connectivity results with DCM demonstrated increased inhibition from the dentate nucleus to the inferior olive. This increased inhibition may represent an increased inhibition of one of the “drivers” of this system (dentate nuclei) that may result in lower functional connectivity or a shift in balance of inhibition/excitation on top of an overall decreased dentato-olivary functional connectivity (baseline). Previously, the inferior olive has also been posited as prime driver of essential tremor mostly because of its pacemaker characteristics. The involvement of the inferior olivary nucleus in essential tremor has been doubted because the evidence is mainly based on neurophysiological recordings in animal studies and there is a lack of evidence in human imaging studies. Also, other structures as the cerebellar nuclei and Purkinje cells have shown pacemaker properties [64, 65]. Our DCM results suggest that the olivo-cerebellar circuit is altered in essential tremor patients. We report increased inhibition from the dentate nucleus to the inferior olive.

### Decreased Connectivity Cerebellar Motor Areas and Thalamus: “Disconnected Motor Cerebellum”

Altogether, our whole-brain analyses point to a “disconnected” cerebellum at rest. This is in line with previous findings [9]. Our results indicate impaired outflow of cerebellar regions, which we specified as possible tremor contributors, at rest. This has previously been shown in two functional imaging studies during action [63]. Lobules VIIIA, both right and left, show bilateral decreased functional connectivity with the thalamus. The thalamus is an important relay hub for cerebellar outflow to the cortex [66]. Lobule VIIIB did not show significant differences in connectivity. In some literature and confirmed by our study, the distinction between lobules VIIIA and VIIIB has proven to be relevant. Where lobule LVIIIA is more associated with motor functions, lobule VIIIB is associated with attentional/executive processes [13].

### Connectivity Related to Sensorimotor Network of the Cerebellum

With our data-driven ICAs, we determined salient networks in the cerebellum and related brainstem structures. We demonstrate this functional division by revealing five cerebellar resting state networks. In addition to the networks that are predominantly associated with sensorimotor functions, others are associated with cognitive functions of the cerebellum including lobules VI, VII, Crus I, and II and ventral part of the dentate nuclei [12, 13]. From these independent components, only those associated with the sensorimotor network were significantly different between groups when back projected to whole-brain maps and not the components that are associated with cognitive functions. The latter further supports the validity of the hypothesis of a “disconnected” motor cerebellum in essential tremor.

### Altered Activity at Rest Leads to Tremor in Action

The main function of the cerebellum and associated brainstem structures regarding motor function is to monitor differences between the intention and implementation of movement, adjusting motor activity, if necessary [67]. Our findings point towards a disbalance of glutamatergic and GABAergic connections leading to inadequate communication between key cerebellar motor structures and the cerebrum. Underlying (subclinical) altered connectivity can impede communication within the cerebellum during action, hampering motor feedforward and feedback control, causing tremor to emerge. In animal studies investigating cerebellar tremor, especially failure of the feedforward control seemed to contribute to tremor [68]. A disturbed timing and organization of antagonistic muscles has been suggested even after interrupting afferent feedback loops. The inferior olive, which normally contributes to motor regulation, might also contribute to the interruption. The inferior olive is coupled to the tremor network via the dentato-rubro-olivary tract, interacting with the cerebello(dentato)-thalamo-cortical network [69]. Our recent electromyography-fMRI study showed inferior olive activity to be correlated with tremor severity in essential tremor (Sharifi et al., submitted 2022).

Altered connectivity can be appointed to mutually nonexclusive theories, including neurodegenerative changes or functional disinhibition. Due to the dynamic nature of tremor (and absence in rest), an involvement of networks with faulty interactions seems more likely rather than a single entity that is continuously pacing. First, it can be explained by a primary cerebellar disorganization (neuronal dysfunction) that might be caused by structural neurodegenerative or disturbed functional integrity leading to a central oscillator. Recent structural studies describe deficits in glutamate climbing fibers, Purkinje cell pathology, and reduced GABAergic currents to the dentate nucleus causing excessive cerebellar oscillations which highlights the importance of the olivo-cerebellar circuit [4, 61]. Computational modeling studies have shown that a loss of GABA_A_ receptors in cerebellar structures causes prolonged decay of currents leading to aberrant oscillations in the olivo-cerebellar circuit [60, 70]. It seems that local abnormalities lead to widespread network involvement when activating the voluntary motor network. In what manner the pathologic activity propagates into the network and the thalamus is still unclear.

Altogether, we report an important role for the olive-cerebellar system in the pathophysiology of essential tremor. There is a decreased connectivity of the olivo-cerebellar system, suggesting an important role for the dentate nucleus. The altered communication in the cerebellum seems to be exposed when connected to the motor loop, which is activated while performing an intentional movement. We hypothesize that during action, disorganized oscillations spread into the motor network, leading to tremor as if “a lid is lifted off the cerebellar jar.”

### Methodological Considerations

Here, our advanced tailored processing techniques focusing on the olivo-cerebellar circuit are proven to be of value. Also, by implementing data-driven ICAs, we were able to investigate this notoriously difficult area because of its size and anatomical location, being prone to artefacts. We performed different analysis methods. We focused on the cerebellum with functional and effective (directional) connectivity using previously defined ROIs and data-driven ICA analyses. With functional connectivity, a dependency between activity of individual brain regions can be determined; however, the underlying causal effects are still unknown. For the dentato-rubro-olivary circuit, we did apply an effective connectivity analysis. The effective connectivity rests on a model (DCM) that has successfully been used to infer directed connectivity between brain regions.

In literature, only a few resting state studies have performed long-distance analyses with predefined regions of interest [29–31]. Unlike our study, significant decreased connectivity was established between the dentate nucleus and cortical areas [29]. In this study, we used SWI to create a dentate nuclei mask, making use of the high iron content of the dentate nucleus. Although we expect that the masks created based on SWI compared with the anatomical mask to overlap and even to give more adequate time courses, we cannot rule out a lower signal-to-noise ratio of our chosen method due to the high iron content of the dentate nucleus. To be able to investigate small areas within the cerebellum and brainstem, higher spatial and temporal resolutions are warranted. This will also help to reproduce our suggested involvement of the inferior olive.

Our ROIs were based on previously defined motor key structures focusing on alterations in the tremor network. Our data-driven analyses, however, identified five cerebellar resting state networks from which four are known to contribute to cognitive processes. In essential tremor, development of cognitive impairment has increasingly been suggested. Future research focusing on cognitive areas in resting state might be of value.

A central issue of essential tremor is its clinical heterogeneity, with perhaps a different underlying pathophysiology that also has implications for research. A homogeneous group is important for our research, especially since resting state imaging does not have a strong contrast to investigate. To tackle this potential problem, we applied stringent inclusion criteria, excluding patients with neurologic signs of any uncertain significance. To further limit variability of essential tremor patients, we only included patients who had a positive family history in the immediate family and reported a positive effect of propranolol on tremor. Our strict selection criteria may affect the comparability of our research with other studies as most studies include (phenotypically) heterogeneous groups.

### Conclusion

Although tremor in essential tremor reveals itself only in action, underlying pathologic processes are already present in the olivo-cerebellar circuit at rest. The olivo-cerebellar circuit seems to be disconnected from the rest of the motor network, leading to an imbalanced coupling within the cerebello-thalamo-cortical network during movement. We hypothesize that during action, aberrant subclinical oscillations are able to spread throughout the motor network, leading to tremor.

### Supplementary Information

Below is the link to the electronic supplementary material.

Supplementary file1 (DOCX 510 KB)
