# Supplementary material for: Differences in Olivo-Cerebellar Circuit and Cerebellar Network Connectivity in Essential Tremor: a Resting State fMRI Study
Source: Cerebellum. 2022 Oct 10;22(6):1123–36. doi: 10.1007/s12311-022-01486-1 (PMC10657290; doi:10.1007/s12311-022-01486-1)
Supplement: Supplementary file 1 — Supplementary file1 (DOCX 510 KB) [file 12311_2022_1486_MOESM1_ESM.docx]

**Supplementary material**

| **#** | **ET /HC** | **Gender** | **Age (yr)** | **Age at Disease onset (yr)** | **TETRAS** | **Presence of**  **voice, face or head tremor** |
| --- | --- | --- | --- | --- | --- | --- |
| **1** | ET | M | 49.7 | <18 | 12.5 | - |
| **2** | ET | F | 80.8 | 60 | 31.5 | Voice, face and head |
| **3** | ET | M | 84.5 | 33 | 36 | Voice, face and head |
| **4** | ET | M | 63.7 | 18 | 23 | Voice |
| **5** | ET | F | 50.0 | <18 | 14 | Voice |
| **6** | ET | M | 64.3 | 20 | 13.5 | Voice |
| **7** | ET | F | 22.5 | 19 | 15 | - |
| **8** | ET | M | 47.5 | 40 | 18 | Voice |
| **9** | ET | M | 53.4 | 16 | 11.5 | Voice |
| **10** | ET | M | 69.4 | 61 | 13.5 | - |
| **11** | ET | M | 63.0 | 43 | 13 | Voice |
| **12** | ET | M | 72.5 | 13 | 28 | Face, head |
| **13** | ET | M | 55.1 | <18 | 16.5 | - |
| **14** | ET | M | 72.1 | 60 | 19.5 | Voice |
| **15** | ET | M | 26.4 | <18 | 11 | - |
| **16** | ET | F | 81.4 | 20 | 34 | Voice, face |
| **17** | ET | M | 43.4 | 30 | 21 | - |
|  | **Median (range)** | M: 13 | 63.0 (22.5-84.5) | 30 (<18 – 60) |  |  |
|  |  | F: 4 |  |  |  |  |
| **1** | HC | F | 26.8 | - | - |  |
| **2** | HC | F | 57.6 | - | - |  |
| **3** | HC | M | 54.3 | - | - |  |
| **4** | HC | M | 66.0 | - | - |  |
| **5** | HC | M | 52.2 | - | - |  |
| **6** | HC | M | 63.4 | - | - |  |
| **7** | HC | M | 55.9 | - | - |  |
| **8** | HC | F | 60.0 | - | - |  |
| **9** | HC | M | 63.5 | - | - |  |
| **10** | HC | M | 24.4 | - | - |  |
| **11** | HC | M | 59.4 | - | - |  |
| **12** | HC | F | 69.8 | - | - |  |
| **13** | HC | F | 51.3 | - | - |  |
| **14** | HC | M | 51.5 | - | - |  |
| **15** | HC | F | 55.4 | - | - |  |
| **16** | HC | M | 63.9 | - | - |  |
| **17** | HC | M | 76.6 | - | - |  |
| **18** | HC | F | 68.7 | - | - |  |
| **19** | HC | M | 78.1 | - | - |  |
|  | **Median (range)** | M: 12 | 59.40 (24.4-78.1) |  |  |  |
|  |  | F: 7 |  |  |  |  |

Table I. Patient characteristic of individual patients in the essential tremor (ET) and healthy control (HC) groups. TETRAS= The Essential Tremor Rating Assessment Scale

TETRAS includes items on action tremor of individual limbs. Upper limb: during “wing beating” posture, finger-nose-finger, forward horizontal reach posture, drawing of Archimedes spiral, handwriting and dot approximation task. Lower limb: raising each limb horizontally and during standing). Also, scoring presence of head, face and voice tremor. All items are rated 0-4.


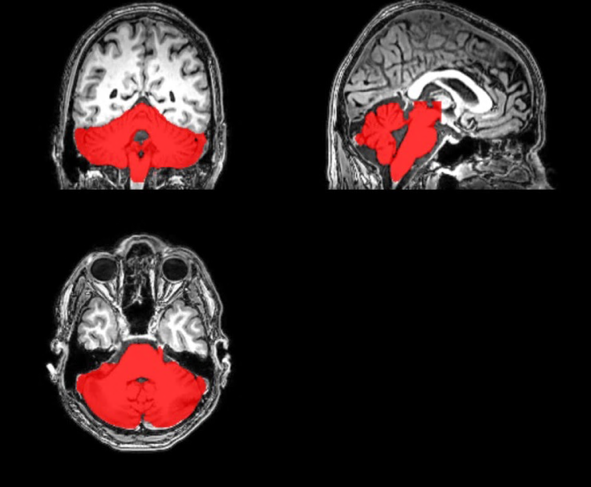

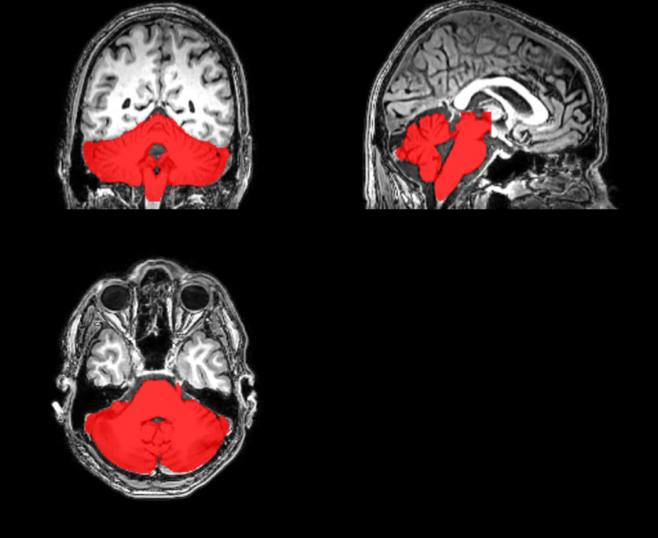


Figure A. Isolation of the cerebellum using the SUIT toolbox.


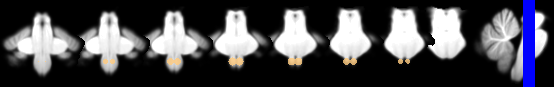


**Figure B**. Inferior olive nucleus mask in yellow based on known coordinates [15,49].


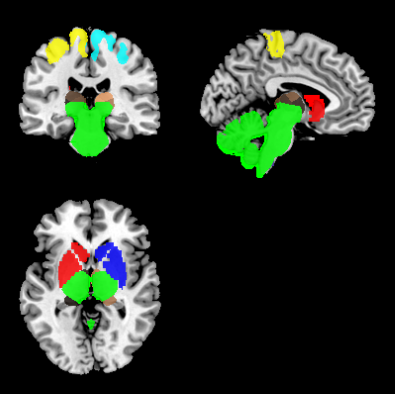


L R

**Figure C**. Region of interest mask in whole brain analyses. Green: cerebellum, red: left basal ganglia, brown: left thalamus, yellow: left sensorimotor cortex.

**Table II.** DCM results for the left olivo-cerebellar circuit

|  | | RN | LV | L8 | DN | ION |
| --- | --- | --- | --- | --- | --- | --- |
| RN | ET  HC | -0.51±0.31  -0.46±0.26 |  |  |  | -0.34 ± 0.8174  -0.38 ±1.0156 |
| LV | ET  HC |  | -0.50± 0.19  -0.62±0.31 | -0.00 ±0.46  -0.09 ±0.28 | 0.08 ± 0.4754  0.07 ±0.4067 |  |
| L8 | ET  HC |  | 0.13 ± 0.67  0.19 ±0.40 | -0.44±.18  -0.59±0.29 | -0.44 ± 0.42  -0.59 ±0.29 |  |
| DN | ET  HC | 0.10 ± 0.48  -0.00 ±0.55 |  |  | -0.48±0.22  -0.56±0.29 | -0.78 ± 0.53  -0.29 ±0.76 |
| ION | ET  HC |  | 0.04 ± 0.13  0.02 ±0.12 | 0.02 ± 0.11  0.02 ±0.11 | 0.10 ±0.17  0.02 ±0.13 | -0.31±0.11  -0.25±0.11 |

**Table III.** DCM results for the right olivo-cerebellar circuit

|  | | RN | LV | L8 | DN | ION |
| --- | --- | --- | --- | --- | --- | --- |
| RN | ET  HC | -0.39±0.15  -0.49±0.29 |  |  |  | -0.43 ±0.5584  -0.41 ±0.9967 |
| LV | ET  HC |  | -0.42±0.15  -0.59±0.30 | 0.13 ±0.45  0.06 ±0.37 | -0.02 ±0.39  -0.05 ±0.35 |  |
| L8 | ET  HC |  | -0.10 ± 0.44  0.07 ±0.42 | -0.39±0.10  -0.49±0.27 | 0.05 ±0.44  -0.16 ±0.36 |  |
| DN | ET  HC | -0.13 ±0.56  0.10 ±0.46 |  |  | -0.48±0.2044  -0.59±0.2456 | -0.42 ±0.7159  -0.16 ±0.7220 |
| ION | ET  HC |  | 0.06 ±0.09  -0.01 ±0.14 | 0.01 ±0.13  0.02 ±0.14 | -0.02 ±0.19  0.00 ±0.13 | -0.30±0.10  -0.24±0.10 |
